# A 26-year-old man with multiple organ failure caused by *Aeromonas dhakensis* infection: a case report and literature review

**DOI:** 10.3389/fmed.2024.1289338

**Published:** 2024-04-17

**Authors:** Dan Luo, Liwan Dai

**Affiliations:** ^1^Department of Respiratory and Critical Care Medicine, People’s Hospital of Chongqing Liang Jiang New Area, Chongqing, China; ^2^Department of Respiratory and Critical Care Medicine, The First Affiliated Hospital of Chongqing Medical University, Chongqing, China

**Keywords:** community-acquired pneumonia, hemoptysis, *Aeromonas*, hemolytic uremic syndrome, multiple organ dysfunction

## Abstract

**Background:**

Infections in humans are mainly caused by *Aeromonas hydrophila*, *Aeromonas caviae*, and *Aeromonas veronii*. In recent years, *Aeromonas dhakensis* has been recognized as widely distributed in the environment, with strong virulence. However, this bacterial infection usually does not appear in patients with pneumonia as the first symptom.

**Case report:**

We report a 26-year-old man who was admitted to the hospital with community-acquired pneumonia as the first symptom and developed serious conditions such as hemolytic uremic syndrome, multiple organ dysfunction, and hemorrhagic shock within a short period. He died after 13 h of admission, and the subsequent metagenomic-next generation sequencing test confirmed the finally identified pathogen of infection as *A. dhakensis*.

**Conclusion:**

*Aeromonas* is a rare pathogen identified in the diagnosis of community-acquired pneumonia. Hence, doctors need to develop their experience in identifying the difference between infections caused by pathogenic microorganisms. Medical attention is essential during the occurrence of respiratory symptoms that could be controlled by empirical drugs, such as cephalosporins or quinolones. When patients with community-acquired pneumonia present hemoptysis and multiple organ dysfunction in clinical treatment, an unusual pathogen infection should be considered, and the underlying etiology should be clarified at the earliest for timely treatment.

## Graphical Abstract

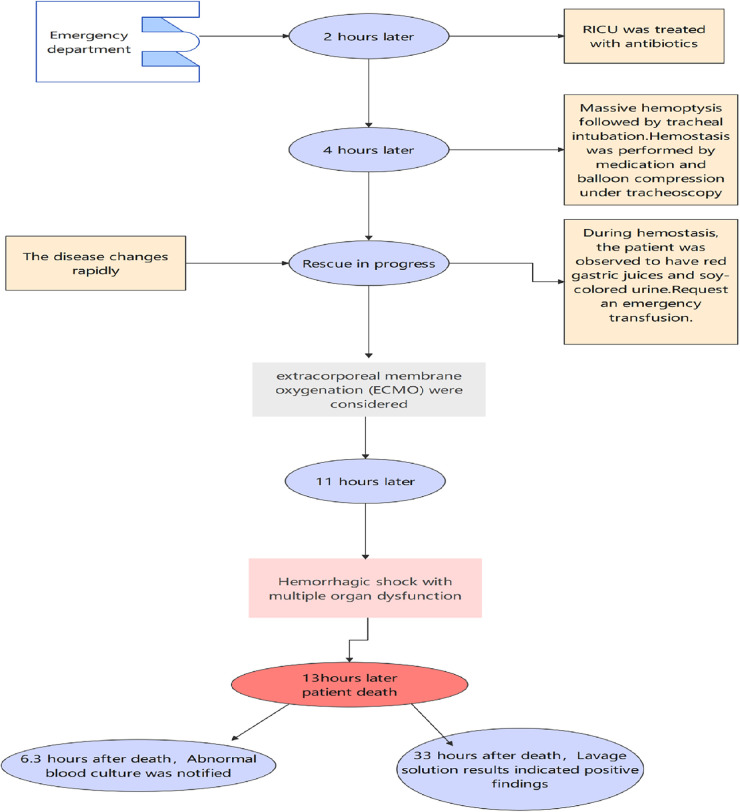

## Introduction

*Aeromonas dhakensis* is a Gram-negative bacillus that is widely distributed in water environments. The mortality rate caused by infection with *A. dhakensis* is higher than that of other *Aeromonas* infections due to the abundance of virulence genes. It causes gastroenteritis, wound infection, sepsis, respiratory tract infection, hepatobiliary disease, urinary tract infection, muscle necrosis, rhabdomyolysis, necrotizing fasciitis, and the rare

hemolytic uremic syndrome. If an acute infection is not treated promptly, it may develop rapidly and lead to serious consequences. In this case study, we reported the onset and treatment of a 26-year-old patient infected with *A. dhakensis*.

## Case report

**FIGURE 1:**
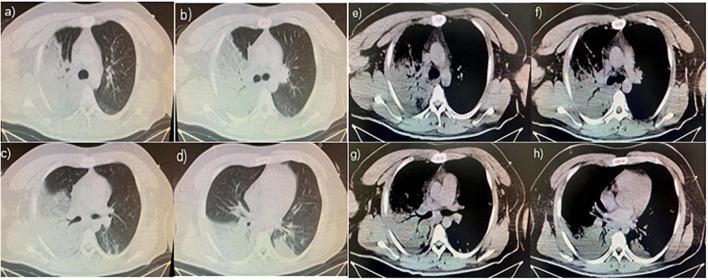
**(a–h)** The chest computer tomography (CT) scan displayed a double lung infection.

A 26-year-old man with no history of lung diseases or other disorders was admitted to the local hospital due to cough and fatigue for 3 days, hemoptysis, dyspnea, fever, chest pain, and wheezing for a day. The patient was admitted to our emergency department. A blood routine examination showed that the white blood cell count was 7.06 × 10^9^/L, and the percentage of neutrophils was 69.9%. The liver function showed alanine aminotransferase at 150 U/L and aspartate transferase at 84 U/L, and the kidney function-related creatinine level was 197 mmol/L, and the uric acid level was 607 mmol/L. The coagulation function D-dimer level was 3730 ng/mL. Blood gas analysis (without oxygen) revealed the following: pH 7.32, PCO_2_ 43 mmHg, PO_2_ 37 mmHg, BE −3.9 mmol/L, HCO_3_ 22.21 mmol/L, Lac 4.8 mmol/L, SO_2_ 65%, Na^+^ 132 mmol/L, K^+^ 3.8 mmol/L, and Glu tendency of 7.1 mmol/L. The Chest computer tomography (CT) scan displayed a double lung infection (Figure 1). A physical examination revealed a body temperature of 37.5°C, and vital signs were within normal range. Lucid, poor spirit, and wet rales could be heard in both lungs. After 2 h, the patient was transferred to the Respiratory Intensive Care Unit (RICU).

According to the examination, the patient was diagnosed with severe pneumonia and abnormal liver function. While being kept on a non-invasive ventilator, 1,000 mg of imipenem was given intravenously every 8 h, 600 mg of linezolid was given intravenously every 6 h, and reduced glutathione, polyene phosphatidylcholine, and carlo sulfonyl sodium were used. After transferring the patient to RICU post 2 h, the hemoptysis level increased to almost 100 ml, and blood gas analysis indicated respiratory failure. Thus, the patient received endotracheal intubation. Then, tracheoscopy was performed and active hemorrhage was observed in the right upper lobe opening. The lavage fluid was collected and cultured. The right upper lobe bronchus was blocked by a bronchoscope balloon, pituitrin, carlo sulfonyl sodium, and hemagglutinin to stop the bleeding.

Typically, the bleeding could be controlled, but it was counterproductive. The blood was drained from the stomach tube, the urine turned to a soy sauce color, and blood pressure dropped. Considering the patient had gastrointestinal bleeding and hemolysis, emergency blood transfusion measures were initiated, and vasoactive drugs were administered. To date, 1,000 ml of blood was aspirated under a bronchoscope, 300 ml from a gastric tube, and 150 ml was drained by the urinary tube, and the patient went into hemorrhagic shock. In addition, antishock treatment and extracorporeal membrane oxygenation (ECMO) were considered.

**FIGURE 2:**
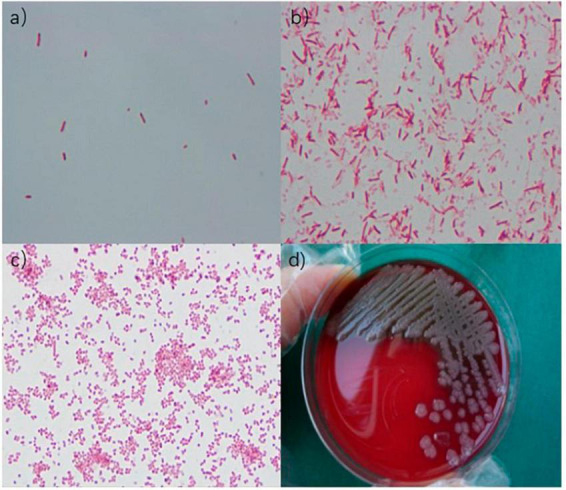
**(a)** Gram-negative bacilli were observed under an oil immersion lens. **(b)** Gram-negative bacilli were observed under an oil immersion lens. **(c)** Gram-negative bacilli were observed under a microscope. **(d)** Medium colony.

These measures stabilized the patient’s blood pressure at 80–95/50–55 mmHg, and the oxygen saturation was stabilized at 80%–90%. Indubitably, the patient’s condition deteriorated rapidly, leading to multiple organ failures before the initiation of ECMO therapy and eventually leading to death. At 6.3 h after death, the blood cultures suggested the presence of *Aeromonas hydrophila/Aeromonas caviae* (Figure 2). At 33 h, irrigation fluid culture also signaled the presence of *A. hydrophila/A. caviae* after the patient’s death. This is the first case of *Aeromonas* infection that we have encountered and also a rare case of multiple organ failure reported in the literature.

## Discussion

*Aeromonas* is a Gram-negative bacillus widely distributed in freshwater, river/estuarine water (brackish water), surface water, drinking water, polluted water bodies, and sewage sludge (1–5). The classification includes *A. hydrophila*, *A. caviae, Aeromonas veronii biovar sobria, Aeromonas milmiliae, Aeromonas salmonicides, Aeromonas intermedialis, Aeromonas janeii, Aeromonas shuiensis*, and *Aeromonas fragilis* (6). Human infections are commonly caused by *A. hydrophila*, *A. veronii biovar sobria*, and *A. caviae* (7). The most common route of infection is contact with fresh or brackish water, which is usually stagnant in warm months (May–October in the Northern Hemisphere), its bacterial count reaches a peak value, thereby elevating the incidence of *Aeromonas* infection in summer (8, 9). Patients with chronic underlying diseases, such as nephritis, diabetes, tumors, leukemia, and hepatobiliary pancreas, have low immunity and are at a high risk of *Aeromonas* infection. It directly enters the blood through the peritoneal barrier and reaches the thoracic tissue, pelvic tissue, lymph, gallbladder, and other parts. In severe cases, it may be life-threatening (10). The patient, in this case, was a 26-year-old man with no underlying diseases or low immunity. However, he had embarked on self-driving trips for 3 days before the onset of the disease. En route, he swam in a lake, which could be the cause of *Aeromonas* infection; the onset was rapid, and the clinical manifestations were critical.

*Aeromonas* cause a variety of human diseases such as diarrhea, wound infection, bacteremia, respiratory tract infection, eye infection, osteomyelitis, meningitis, pelvic abscess, otitis, cystitis, endocarditis, peritonitis, cholecystitis, joint infection, necrotizing fasciitis, and folliculitis (11–16). Diarrhea is the most common clinical manifestation. Different from the typical infection symptoms, this patient had respiratory symptoms of cough, expectoration, and hemoptysis as initial presentation, without diarrhea.

Within 11 h of admission, 1,000 ml of blood was aspirated under a bronchoscope, 300 ml of blood from the gastric tube, and 150 ml of blood was drained by the urinary tube. Subsequently, *Aeromonas* was detected in blood culture and bronchoscopic lavage fluid culture, and the biochemical results are shown in Table 1. The results indicated that the patient was infected with *Aeromona*s, leading to *Aeromonas* hemolytic-uremic syndrome, which is a rare manifestation caused by this bacterium. Since *A. hydrophila/A. caviae* is often confused with *A. dhakensis* due to similar homology, conventional automatic laboratory biochemical identification cannot distinguish the species type effectively (17). Hence, metagenomic-next generation sequencing was recommended for species identification by whole genome sequencing. The results finally confirmed *A. dhakensis* infection in the patient.

**TABLE 1:** Biochemical identification of *A. dhakensis*.

Biochemical details																	
2	APPA	+	3	ADO	r	4	PyrA	−	5	IARL	−	7	dCEL	+	9	BGAL	+
10	H2S	−	11	BNAG	+	12	AGLTp	−	13	dGLU	+	14	GGT	+	15	OFF	+
17	BGLU	+	18	dMAL	+	19	Dman	+	20	dMNE	−	21	BXYL	−	22	Balap	−
23	ProA	+	26	LIP	+	27	PLE	−	29	TyrA	+	31	URE	−	32	dSOR	−
33	SAC	+	34	dTAG	−	35	dTRE	+	36	CIT	−	37	MNT	−	39	5KG	−
40	ILATk	+	41	AGLU	−	42	SUCT	+	43	NAGA	−	44	AGAL	−	45	PHOS	−
46	GlyA	−	47	ODC	−	48	LDC	−	53	IHISa	−	56	CMT	+	57	BGUR	−
58	O129R	+	59	GGAA	+	61	IMLTa	−	62	ELLM	+	63	ILATa	−			

APPA, ala-phe-pro-arylamidase; H2S, H2S production; BGLU, β-glucosidase; ProA, L-proline arylamidase; SAC, saccharose; ILATk, L-lactate alkalinization; GlyA, glycine arylamidase; O129R, O/129 resistance; ADO, adonitol; BNAG, β-N-acetyl-glucosaminidase; dMAL, d-maltose; LIP, lipase; dTAG, d-tagatose; AGLU, α-glucosidase; ODC, ornithine decarboxylase; GGAA, glu-gly-arg-arylamidase; PyrA, L-pyrrolidonyl-arylamidase; AGLTp, glutamyl arylamidase Pna; Dman, D-mannitol; PLE, PALATINOSE; dTRE, d-trehalose; SUCT, succinate alkalinization; LDC, lysine decarboxylase; IMLTa, L-malate assimilation; IARL, L-arabitol; dGLU, d-glucose; dMNE, d-mannose; TyrA, tyrosine arylamidase; CIT, citrate; NAGA, N-acetyl-β-galactosaminidase; IHISa, L-histidine assimilation; ELLM, ELLMAN; dCEL, d-cellobiose; GGT, γ-glutamyl-transferase; BXYL, β-xylosidase; URE, urease; MNT, malonate; AGAL, α-galactosidase; CMT, COURMARATE; ILATa, L-lactate assimilation; BGAL, β-galactosidase; OFF, fermentation glucose; Balap, β-alanine arylamidase; dSOR, d-sorbitol; 5KG, 5-keto-glucoside; PHOS, phosphatase; and BGUR, β-glucuronidase.

It is a subspecies of *A. hydrophila*, also known as *Aquariumonas*. It was originally isolated from children with diarrhea in Bangladesh during the period of 1993–1994 (18). In Beaz-Hidalgo et al. (19), ascertained that the *Dakar* subspecies of *A. hydrophila* and *A. aquarium* were the same species and that the *Dakar* subspecies were different from other subspecies of *A. hydrophila*. Therefore, the *Dakar* subspecies of *A. hydrophila* and *A. aquarium* were merged into a new species of *A. dhakensis* (19). Accumulating evidence shows that *A. dhakensis* is widely distributed in the environment, primarily in coastal areas, and can cause various human and animal infections, including gastroenteritis, wound infection, sepsis, respiratory tract infection, hepatobiliary disease, urinary tract infection, muscle necrosis, rhabdomyolysis, and necrotizing fasciitis (20). The reported mortality rate among patients with *A. dhakensis* extraintestinal infection varies from 25.5% to 37.5%, which is much higher than in those infected with other *Aeromonas* species (0%–14%) (20, 21). Previous studies reported that *A. dhakensis* carries several virulence factors and exerts high toxicity on human blood cell lines (22–25) via exotoxins (act, aerA, hlyA, alt, ast, and other genes), type III secretion system (aexT, aopP, ascF-G, ascV, and other genes), extracellular enzymes (gcat, exu, ahyB, lip, ser, epr CAI, and other genes), adhesion factors associated with invasion (tapA gene), and flagella (laf and fla genes) (7). The high mortality rate and the abundance of virulence genes make it a crucial pathogenic species. However, the pathogenesis mechanism and regulation of toxicity remain unclear (26). The majority of *Aeromonas* bacteria carry at least one virulence gene. Act, hlyA, aerA, gcat, and lip genes related to cytolysis were detected in both enteric *Aeromonas* and exenteric *Aeromonas* to varying degrees, which cause hemolysis in the body.

*Aeromonas dhakensis* is susceptible to cefepime, aminoglycosides, fluoroquinolones, and tetracyclines, and hence, these drugs can be used for the treatment of the infection (22, 27). Chao et al. (28) indicated that more than 80% of clinical strains are sensitive to third- or fourth-generation cephalosporins, aminoglycosides, fluoroquinolones, and imipenems. Therefore, the above drugs are still the first choice for treatment. However, *A. dhakensis* intrinsically harbors class B (metallo-β-lactamases, MBLs; CphA), C (AmpC cephalosporinase; AQU-1), and D β-lactamases (penicillinases). CphA has a specific substrate profile for hydrolyzing carbapenems; however, carbapenem therapy using CphA on *Aeromonas* infections remains controversial (29, 30). It is notable that ertapenem is a breakthrough in the treatment of bacteremia caused by *A. dhakensis* (29). The best antibacterial options for treating *A. dhakensis* infection include fluoroquinolone or cefepime until susceptibility results are available (20). For severe pneumonia cases, we chose imipenem and linezolid for anti-infection treatment. Combined with blood culture and drug sensitivity test of the patient as shown in Table 2, *A. dhakensis* was found to be susceptible to ceftazidime (MIC = 2 μg/ml), cefoperazone/sulbactam (MIC = 16 μg/ml), cefepime (MIC = 0.5 μg/ml), aztreonam (MIC ≤ 1 μg/ml), imipenem (MIC ≤ 0.25 μg/ml), meropenem (MIC ≤ 0.25 μg/ml), amikacin (MIC ≤ 2 μg/ml), ciprofloxacin (MIC ≤ 0.25 μg/ml), levofloxacin (MIC ≤ 0.12 μg/ml), tigecycline (MIC ≤ 0.5 μg/ml), and trimethoprim/sulfamethoxazole (MIC ≤ 20 μg/ml). *A. dhakensis* was found to be resistant to piperacillin/tazobactam (MIC ≥ 128 μg/ml). The drug resistance genes of the bacteria could not be ascribed to any carbapenemase genes (CphA, KPC, IMP, VIM, NDM, and OXA-48-like), extended-spectrum β-lactamase genes (CTX-M-1, CTX-M-9, CTX-M-15-like, TEM, and SHV), and Ampc enzyme genes (ACC and FOX). The pathogenic bacterial infection was susceptible to imipenem cilastatin sodium. Unfortunately, the patient died due to rapid disease progression.

**TABLE 2:** Drug sensitivity test of the patient.

Susceptibility information	Analysis time: 7.37 h			Status: final	
Antimicrobial	MIC	Interpretation	Antimicrobial	MIC	Interpretation
Ticarcillin/clavulanic acid			Tobramycin		
Piperacillin/tazobactam	≥128	R	Ciprofloxacin	≤0.25	S
Ceftazidime	2	S	Levofloxacin	≤0.12	S
Cefoperazone/sulbactam	16	S	Doxycycline		
Cefepime	0.5	S	Mnocycline		
Aztreonam	≤1	S	Tigecycline	≤0.5	S
Imipenem	≤0.25	S	Colistin		
Meropenem	≤0.25	S	Trimethoprin/sulfamethoxazole	≤20	S
Amikacin	≤2	S			

## Conclusion

Based on the diagnosis and treatment process of this patient, we realized that *Aeromonas* is not a common bacterium in doctors’ empirical diagnoses of community-acquired pneumonia patients. First, the pathogenic microorganisms responsible for hemoptysis in community-acquired pneumonia are *Staphylococcus aureus*, *Mycobacterium tuberculosis*, *Streptococcus pneumoniae*, *Klebsiella pneumoniae*, *Aspergillus*, and *Mucor*. *Aeromonas*, and infection with community-acquired pneumonia as the initial symptom and hemolysis lead to pulmonary hemorrhage, multiple organ dysfunction, and hemorrhagic shock, is rare. Second, the patient was admitted to the hospital with respiratory symptoms rather than the common symptom of diarrhea caused by *Aeromonas* infection, which is a rare manifestation of *Aeromonas* infection, and the occurrence of hemolytic uremic syndrome is again a rare clinical symptom. Third, based on the literature review, *Aeromonas* infection occurs in individuals with low immunity to the disease (31); however, this patient was young with no history of basic diseases or low immunity situation. In the present case, *Aeromonas* caused rapid progression, indicating that it could also infect the normal immune population and can also appear in severe manifestations. Fourth, although the patient had a clear manifestation of hemolytic uremic syndrome caused by *Aeromonas* infection, *Escherichia coli* is the most common pathogen causing the syndrome, followed by *Shigella* dysentery, *Salmonella*, *Campylobacter*, *Yersinia*, and enterovirus (32, 33). Only two cases of *Aeromonas* causing hemolytic uremic syndrome have been reported to date (33–35), and hence, the clinical presentation of this patient is rare. Fifth, if the patient was admitted to the hospital when respiratory symptoms occurred, cephalosporins or quinolones were administered according to the experience of doctors in the treatment of community-acquired pneumonia in order to make a life-saving treatment attempt.

## Data availability statement

The original contributions presented in this study are included in the article, further inquiries can be directed to the corresponding author.

## Ethics statement

The studies involving humans were approved by the First Affiliated Clinical Research Ethics Review Committee of Chongqing Medical University. The studies were conducted in accordance with the local legislation and institutional requirements. The participants provided their written informed consent to participate in this study. Written informed consent was obtained from the relation for the publication of any potentially identifiable images or data included in this article.

## Author contributions

DL: Writing – original draft, Writing – review & editing. LD: Writing – original draft, Writing – review & editing.
